# Comparison of Osseointegration of Dental Implants Placed in Rabbit Tibia Using Two Dental Laser and Implant Handpiece Systems

**DOI:** 10.3390/bioengineering9110681

**Published:** 2022-11-11

**Authors:** Jin-Han Park, Keunbada Son, Young-Tak Son, Yong-Gun Kim, Jo-Young Suh, Kyu-Bok Lee, Jae-Mok Lee

**Affiliations:** 1 Department of Periodontology, School of Dentistry, Kyungpook National University, Daegu 41566, Korea; 2 Advanced Dental Device Development Institute (A3DI), Kyungpook National University, Daegu 41566, Korea; 3 Department of Dental Science, Graduate School, Kyungpook National University, Daegu 41566, Korea; 4 Department of Prosthodontics, School of Dentistry, Kyungpook National University, Daegu 41566, Korea

**Keywords:** dental implant, laser, implant stability, osseointegration

## Abstract

The present study aimed to confirm the usefulness of a multi-laser handpiece system currently under development. Implants were placed in the tibia of rabbits using a conventional separate laser-implant handpiece system (control group; SurgicPro+; NSK, Kanuma, Japan and Epic 10; Biolase, Irvine, CA, USA) and a multi-laser handpiece system (experimental group; BLP 10; Saeshin, Daegu, Korea). Implants were placed in left and right tibias of five rabbits using a conventional laser-implant handpiece system and a multi-laser handpiece system (N = 5 per group). Subsequently, micro-computed tomography (micro-CT; bone-to-implant contact evaluation), implant stability quotient (ISQ) measurement, and histological evaluations were performed to confirm the implant placement results. The independent t-test and the paired *t*-test were used to compare the ISQ values and the results of the two implant-laser handpiece groups (α = 0.05), respectively. No statistically significant difference in micro-CT, ISQ, and histological evaluations was observed between implant placement by the two systems (*p* > 0.05) except implant initial stability. The use of the multi-laser handpiece system is expected to produce the same results as a conventional separate laser-implant handpiece system with the higher implant initial stability. Additionally, it will potentially make the clinical environment more pleasant and will provide convenience for the clinicians.

## 1. Introduction

After the development of light amplification via the stimulated emission of radiation (laser) by Maiman in 1960 [1], several scholars have tried to apply laser clinically, starting with the medical use of lasers by Goldman in 1961 [2]. The various uses of laser have recently emerged in the dental field, where laser systems can make surgery more convenient, both for the patient as well as the operator [3]. The irradiation of soft tissue with a laser can induce an atraumatic cut and allow homogenous tissue removal. Although the existing scalpels cannot perform hemostasis and incision simultaneously [4,5], it can be performed separately or simultaneously using a laser, which reduces discomfort after surgery [6]. Lasers can also be used in periodontal treatment. While traditional mechanical treatments, such as scaling and subgingival curettage, do not significantly affect bacteria such as *Aggregatibacter actinomycetemcomitans* [7,8,9], lasers can effectively reduce the number of bacteria without damaging the subgingival tissue [10,11,12]. A certain amount of irradiated laser is absorbed by the periodontal tissue and converted into energy, which can play a role in accelerating the regeneration process of periodontal tissue [13]. Numerous types of lasers are currently available in the market, and each has its own indications [14]. The neodymium: yttrium aluminum garnet (Nd: YAG) laser operates at a wavelength of 1064 nm and is mainly absorbed by pigmented tissue; it can be used for subgingival curettage, desensitization, biopsy, aphthous ulcer treatment, and peri-implantitis treatment [15,16,17]. The erbium: yttrium aluminum garnet (Er: YAG) laser operates at a wavelength of 2940 nm, has high affinity for water, and reacts strongly with hydroxyapatite to remove enamel and dentinal caries [18,19]. Gingivectomy, gingivoplasty, frenectomy, and laser peeling can be performed using a CO_2_ laser [20,21,22]. The diode laser can be used to perform soft tissue surgery in the continuous or pulsed mode at a wavelength of 810–980 nm [12,23,24]. The diode laser is replacing the incision method using the traditional scalpel. According to a recent study, when soft tissue incision was made using scalpel, traditional diode laser and a multi-laser handpiece system, histological and immunohistochemical pattern of soft tissue healing were not significantly different [25]. Using the photothermal effect, the predicate or oral mucosa was excised or subjected to ablation/vaporization.

With aging of the population, implant treatment has become more popular; soft tissue treatment using a laser prior to dental implant placement, and the use of an implant handpiece for implant placement, have become widespread [26,27,28]. When a conventional separate laser-implant handpiece system is used, in which the laser device and the implant handpiece are used as independent devices, the congestion in the operating room increases, which may cause discomfort to operators, assistants, and the patient. A multi-laser handpiece system integrating a diode laser and an implant handpiece into one device was recently developed to address these problems. If the clinical results of implant placement procedure show no significant difference, it is potentially expected that the use of a multi-laser handpiece system that integrates the two machines into one device would improve the clinician’s convenience and provide a pleasant environment for the clinic. Therefore, the present study aimed to investigate the differences in clinical results after placing implants in the tibia of rabbits using a conventional separate diode laser-implant handpiece system, and a multi-laser handpiece system. The null hypothesis of the present study was that there is no significant difference in the degree of osseointegration of implants placed using two handpiece systems.

## 2. Materials and Methods

### 2.1. Experiment Preparation

The research protocol of the present study was reviewed and approved by the Institutional Animal Care and Use Committee of the Laboratory Animal Center of Daegu Gyeongbuk Medical Innovation Foundation (approval number: DGMIF-21040804-00).

**Figure 1:**
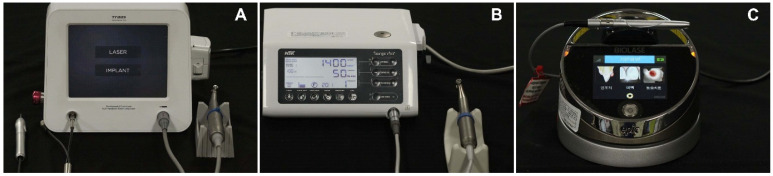
Handpiece system. (**A**) Multi-laser handpiece system (Experimental group). (**B**) Conventional implant handpiece system (control group). (**C**) Conventional laser handpiece system (control group).

**Figure 2:**
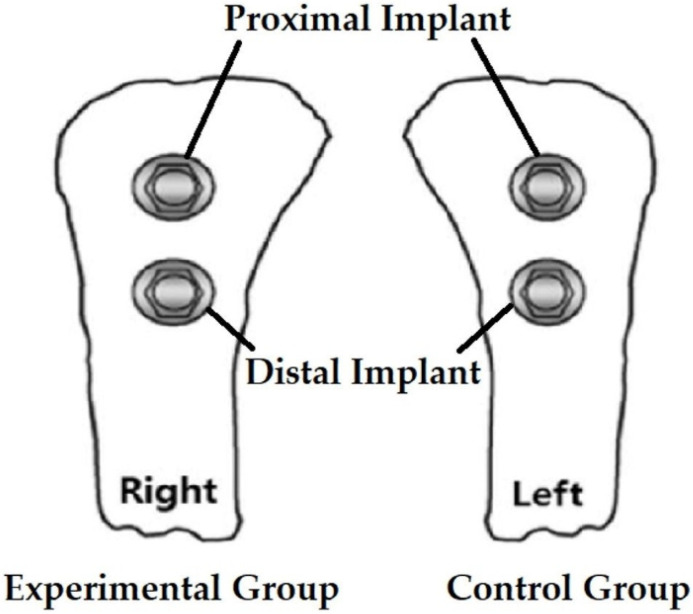
Implant placement sites in rabbit tibia.

Implants were placed in tibias of five rabbits using a conventional laser-implant handpiece system and a multi-laser handpiece system. Two implants were placed in the right tibia of each rabbit in the experimental group (BLP 10; Saeshin, Daegu, Korea, multi-laser handpiece system) (Figure 1A), and two implants were placed in the left tibia of each rabbit in the control group (SurgicPro+; NSK, Kanuma, Japan (Figure 1B) and Epic 10; Biolase, Irvine, CA, USA (Figure 1C); conventional laser-implant handpiece system); the implant placement sites are shown in Figure 2. Four weeks after implant placement, the rabbits were sacrificed, and the clinical results of implant placement were compared using micro-CT (Quantum FX; PerkinElmer Health Sciences, Hopkinton, MA, USA), implant stability quotient (ISQ) values (Osstell^®^ Beacon device; W&H, Göteborg, Sweden), and histological evaluation (bone-to-implant contact (BIC) ratio).

### 2.2. Surgical Procedure

**Figure 3:**
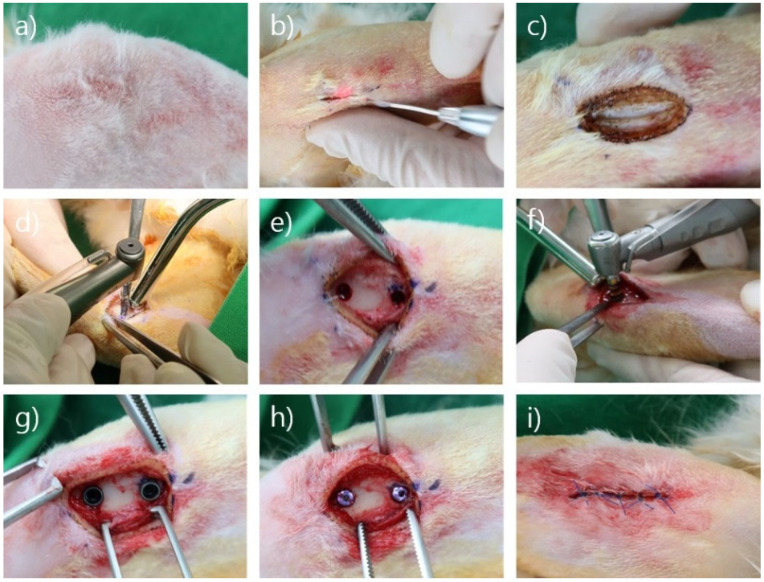
Surgical procedure. (**a**) Hair removal. (**b**) Incision with a laser. (**c**) Flap elevation. (**d**) Drilling of the implant hole. (**e**) After drilling the implant hole. (**f**) Insertion of the implant fixture. (**g**) Implant fixture placement. (**h**) Cover screw adaptation. (**i**) After suturing.

Anesthesia (ketamine, 35 mg/kg; Rompun 5 mg/mL) was administered intramuscularly, followed by inhalational anesthesia (isoflurane 1–2%). After hair removal around the proximal tibia, skin incisions were made using each diode laser (continuous wave mode, 1.5 W; experimental group wavelength, 980 nm; control group wavelength, 940 nm). After making a hole using the initial drill, 2.8-mm shaping drill, and 3.3-mm shaping drill, the implant fixture was inserted using each implant handpiece and torque ratchet (AnyOne fixture; internal type; diameter, 3.5 mm; length, 7.0 mm; Megagen, Daegu, Korea). The ISQ of each implant was measured and the cover screw was fastened. After suturing, the specimens were sterilized using povidone, and a collar was placed around the neck until the sutured area was healed completely (Figure 3).

### 2.3. ISQ, Bone-to-Implant Contact (BIC), and Histological Evaluation

**Figure 4:**
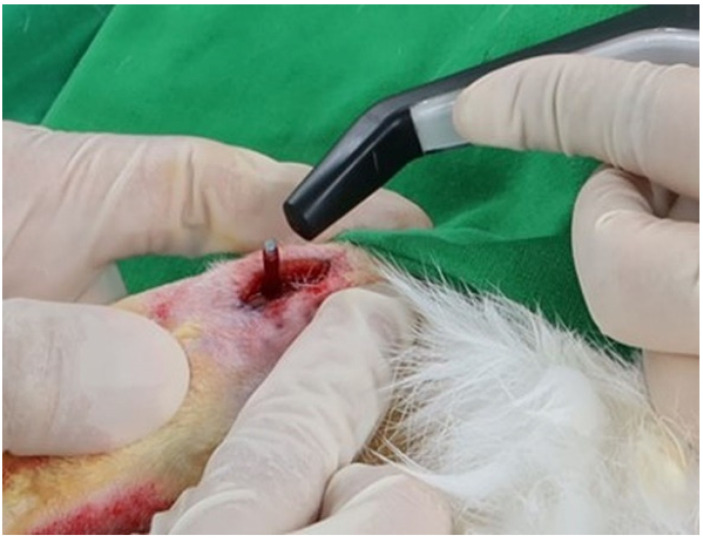
Measurement of the implant stability quotient immediately after surgery.

**Figure 5:**
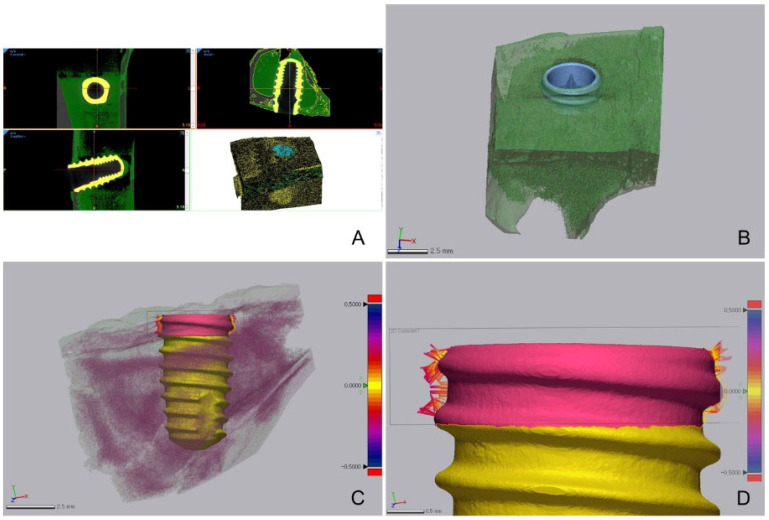
Procedure for analyzing the bone-implant-contact (BIC). (**A**) Segmentation of the implant and bone tissue. (**B**) Segmented implant and bone tissue. (**C**,**D**) BIC analysis in the cortical bone area. Pink indicates cortical bone, while yellow indicates cancellous bone (from yellow to purple, the distance is farther).

After four weeks, the rabbits were sacrificed and the ISQ of each implant was measured (Figure 4). After ISQ measurement, only the tibia was isolated from the subject, and micro-CT was performed (Quantum FX micro CT; Perkin Elmer) (Figure 5). Data acquired using micro-CT were converted into a 3D model using 3D surface modeling software (Mimics; Materialise, Leuven, Belgium) (Figure 5). Then, the implant and bone tissue were segmented by adjusting the intensity level. In order to avoid measuring BIC in cancellous bone, the cortical bone area was separated from the isolated implant and bone tissue (Figure 5). Then, the BIC was analyzed by measuring the distance between the surface of implant and bone tissue in the cortical bone area by using a 3D model (Figure 5).

Each tibia was fixed with 10% formalin for histological evaluation. The fixed sample was immersed in Villanueva bone stain solution for one week after washing. After dehydrating the tissue sample, it was immersed in a methyl methacrylate monomer and then embedded using methyl methacrylate embedding medium resin. After curing at 37 °C, a microcutter (Maruto, Japan) was used to cut the implant fixture to a thickness of 400 µm and attach it to an acrylic slide. After grinding, 40–60 µm thick specimens were prepared, and the differences in BIC were compared by using cross-sectional specimens.

### 2.4. Statistical Analysis

All the acquired data were verified for normal distribution using statistical analysis software (IBM SPSS Statistics v25.0; IBM Corp., Armonk, NY, USA), and the normal distribution was confirmed (α = 0.05). The independent t-test and the paired t-test were used to compare the ISQ values and the results of the two implant-laser handpiece groups (α = 0.05), respectively.

## 3. Results

### 3.1. Comparison of Soft Tissue Healing after Laser Incision

**Figure 6:**
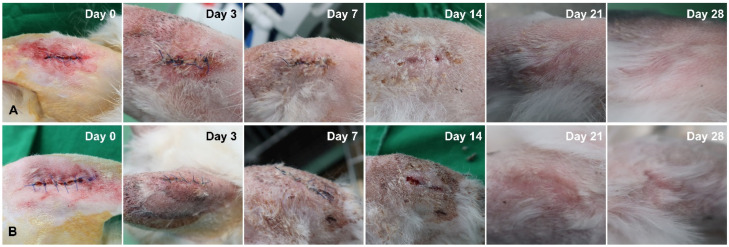
Comparison of soft tissue healing after laser incision between two groups. (**A**) Multi-laser handpiece system (experimental group). (**B**) Conventional laser handpiece system (control group).

The tissues in all subjects showed good healing, without any complications. Visual examination was performed by one inspector, who confirmed that soft tissue healing was achieved in both groups by the 28th day (Figure 6). Additionally, no difference in healing was found in the experimental and control groups (Figure 6).

### 3.2. Comparison of BIC Obtained Using Micro-CT

After implant placement using each system, the bone volume ratio (bone volume/tissue volume; BV/TV), BIC ratio, and bone-to-implant distance around the implant were measured using micro-CT imaging. For bone volume ratio measurement, the range was limited to the cancellous bone.

Four weeks after implant placement, the distribution of bone tissue on the implant surface (BV/TV ratio excluding the cortical bone) was measured. The BV/TV ratio in the control group was 2.85% (proximal, 2.85%; distal, 2.85%), while that in the experimental group was 3.05% (proximal, 3.14%; distal, 2.95%). The two groups showed no statistically significant differences (Table 1).

**Figure 7:**
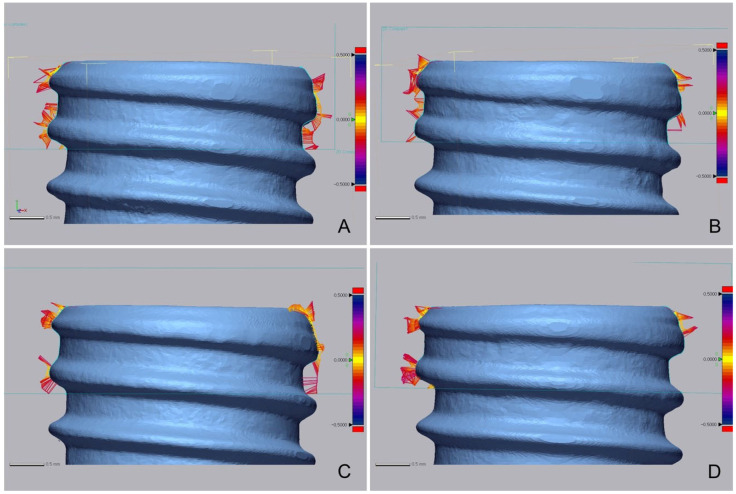
Comparison of bone-to-implant contact images. (**A**) Control group (proximal implant). (**B**) Control group (distal implant). (**C**) Experimental group (proximal implant). (**D**) Experimental group (distal implant).

Micro-CT data were processed using 3D medical image processing software (Mimics; Materialize, Leuven, Belgium) to separate the implants and bone tissue. After separating the implant and bone tissue, 3D analysis software (Geomagic control X; 3D systems, Rock Hill, CA, USA) was used to measure the distance between the implant and the bone and the BIC ratio (%) in the cortical bone area (Table 2 and Table 3, Figure 7).

The BIC ratio was 44.69% in the experimental group and 48.78% in the control group. The two groups showed no statistically significant difference in BIC ratio using micro-CT. The micro-CT measurements of the distance between the implant and the bone showed no statistically significant difference between the two groups.

### 3.3. Comparison of ISQ Values

Using each system, the ISQ of each implant was measured immediately after placement and one month after implantation. The ISQ was measured six times per implant, three times each on the buccolingual and mesiodistal surfaces.

Significant intragroup differences were found in the ISQ values immediately after implant placement and one month after implant placement in both the experimental and control groups. Immediately after implant placement, the experimental group showed a significantly higher ISQ value (mean, 70.1 ± 10.4) than that of the control group (mean, 65.2 ± 11.6); however, one month after implant placement, the experimental group showed a mean value of 79.9 ± 4.9, while the control group showed a mean value of 79.0 ± 4.5, indicating no statistically significant intergroup difference (Table 4).

### 3.4. Comparison of Histological Results

**Figure 8:**
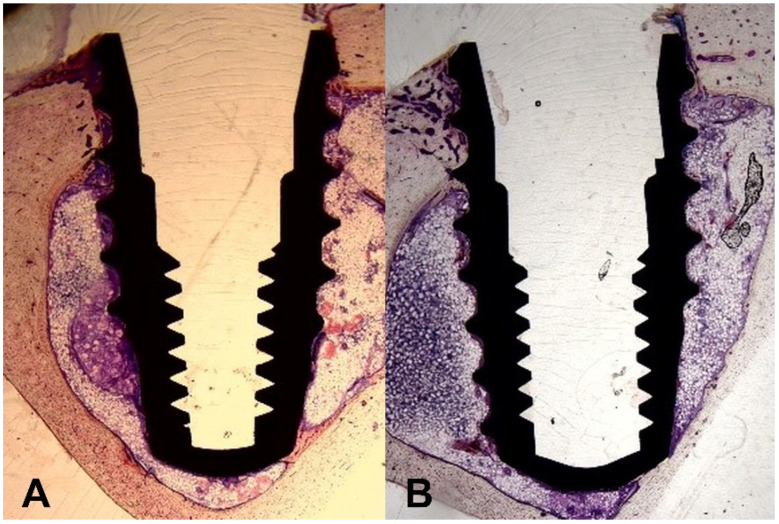
Comparison of histological results. (**A**) Multi-laser handpiece system (experimental group). (**B**) Conventional handpiece system (control group).

Histological evaluation was performed with three samples from each group. In the experimental group, three samples were prepared only from the distal side of the implant in the rabbit tibia, while in the control group, one sample was prepared from the proximal side of the implant and two from the distal side of the implant in the rabbit tibia (Figure 8). On the basis of the histological results, the BIC ratio was compared.

The mean of BIC ratio was 55.52% in the experimental group and 59.06% in the control group. The two groups showed no statistically significant difference in histological BIC ratio (Table 5).

## 4. Discussion

The purpose of the present study was to investigate the differences in clinical results after placing implants in the tibia of rabbits using a conventional separate diode laser-implant handpiece system, and a multi-laser handpiece system. The null hypothesis was that there is no significant difference in the degree of osseointegration of implants placed using two different handpiece systems. Based on the study results, the null hypothesis was accepted (*p* > 0.05; Table 1, Table 2, Table 3, Table 4 and Table 5).

Micro-CT analysis showed no significant difference in the BV/TV ratio between the two groups, indicating no difference in the bone volume ratio between the two groups. Moreover, the BIC ratio did not differ between the two groups, implying that osseointegration was properly achieved in both groups. Compared to other studies, the BV/TV ratio was lower (32.92% by Kfouri et al. [29], 28.66% by Barak et al. [30] and 26.28% by Al-Mahalawy et al. [31]), and the BIC ratio was similarly measured (52.87% by Al-Mahalawy [31]). Although BV/TV ratio was lower than other researches due to the fact that, when setting the range for measuring the BV/TV ratio, the TV range was broadened, it is meaningful in that there was no significant difference between the two groups within the present study, which means similar bone volume was gained. The fact that the BIC ratio value was measured similarly to other studies can be interpreted that a similar degree of osseointegration was formed in the present study. In the present study, the distance between the surface of implant and bone tissue in the cortical bone area was measured using a 3D model. As far as the authors of the present study are aware, it is difficult to compare the results of this study with other literature because bone-to-implant distance measurements have not been performed in other literature. However, distance measurement was necessary to confirm proper bonding between the implant and bone, and in the present study, the distance between the implant and bone did not show any intergroup difference, indicating proper bonding between the implant and bone in both groups.

ISQ measurement is a reliable, accurate, and non-invasive method most commonly used to measure implant stability clinically. Both groups showed significant intragroup differences in the ISQ values measured immediately after implant placement, and at one month after implant placement, indicating that osseointegration progressed adequately at one month after implant placement in both groups. The ISQ values measured after 6 weeks were similar to those of other studies (68~70 by Yeo et al. [32] and 73~78 by Rios-Carasco et al. [33]), and through this, proper implant stability to withstand loading was achieved. Immediately after surgery, a higher ISQ value was noted in the experimental group than in the control group; however, at one month after surgery, both groups showed similar ISQ values. This suggests that although the initial fixation was better in the experimental group, osseointegration progressed well in both groups, with no significant intergroup differences.

Although the measurement of implant stability through micro-CT or ISQ is non-invasive, it has the disadvantage that osseointegration cannot be confirmed as directly as histological investigation. Histological BIC ratio in the present study was similar to that of other studies (35.16% by Kfouri et al. [29], 45.2% by Yeo et al. [32] and 59.9% by Rios-Carrosco et al. [33]), and histological observations confirmed that proper osseointegration was gained, directly. However, compared to micro-CT or ISQ measurement, histological observation has a limitation that the result value may be different depending on which part is used as a sample, so the results should be interpreted with caution. In the histological comparison, the difference in BIC ratio between the two groups was not significant. The BIC ratio obtained using the histologic method was higher than that obtained using micro-CT, because the area could be more precisely identified in the cortical bone histologically. As a result, it showed more precise and higher values than those seen with micro-CT.

The soft tissues healed properly in both groups, and no complications, including swelling, inflammation, and dehiscence, were observed during the healing process. This experiment had a limitation in that no tests were performed that could quantify the significance of two methods, such as immunological parameters and histological examination. In a recent study, the histological and immunohistochemical results of surgical wounds treated using a conventional steel scalpel, and a diode laser of a multi-laser handpiece system, were analyzed. According to that study, the difference of histological and immunohistochemical pattern of healing between two groups did not exist. In addition, there are research results that conclude that incision using a diode laser reduces the discomfort of patients during surgery and reduces bleeding, compared to the method using a scalpel. It was indicated that the diode laser of a multi-laser handpiece system is expected to replace the traditional scalpel when performing incisions on soft tissue [25]. Likewise, in the present study, both groups obtained adequate stability of the implant, which inferred that the superior soft tissue healed adequately enough to have no effect on the inferior hard tissue. In addition, there is a study that confirmed the healing pattern up to 28 days after making an incision in rabbit skin using a diode laser of 1.5 W, 3.0 W, and 5.0 W power, respectively. In the group using the diode laser of 1.5 W power, the incision time was the longest, but the tissue damage was the least, showing rapid clinical and histological healing response compared to other groups [34,35]. The diode laser in the present study is 1.5 W power, and it can be inferred that it is more advantageous for soft tissue healing than the 3.0 W and 5.0 W power diode lasers.

The degrees of osseointegration in both groups were estimated through various methods, including micro-CT, ISQ, and histological examination. In both groups, proper primary stability was obtained immediately after the implant placement, and the implant’s stability of withstanding loading was also obtained after the completion of the healing process. The above results were consistent with the existing implant success criteria [36,37,38]. The ISQ value of implants placed by a multi-laser handpiece system measured immediately after the implant placement was significantly higher than that of the conventional separate handpiece system, meaning the multi-laser handpiece system achieved a higher level of primary stability than a conventional separate handpiece system. Obtaining a primary stability is essential to acquire overall implant stability, because it offers proper support for secondary stability, during the bone remodeling process [39]. Therefore, in the circumstance of difficulty in acquiring a proper primary stability, such as poor bone quality, a multi-laser handpiece system may be considered over a conventional separate handpiece system. After healing was completed, no significant difference was found between the conventional separate handpiece system and the multi-laser handpiece system, meaning that similar levels of osseointegration were obtained via two systems. Overall, similar levels of mechanical and biological performance were achieved in two different systems: the multi-laser handpiece system, and a conventional separate handpiece system.

Through this study, it was confirmed that degree of osseointegration was similar to the results of other studies. The multi-laser handpiece system combines both the properties of lasers and implant motor system, suggesting it could better offer simplified surgical procedures and clinical environment. As there were no statistically significant differences between the performance results of the two systems, the multi-laser handpiece system is likely more convenient for clinical application. In addition, the use of a 1.5 W power diode laser is expected to reduce patient discomfort and promote rapid soft tissue healing [40].

However, the limitation of this study is the small size of subjects. Additionally, as this study was tested on animals, additional clinical trials including patient-centered assessments are required.

## 5. Conclusions

There was no statistically significant difference between the two groups when implants were placed using the conventional separate laser-implant handpiece system and the multi-laser handpiece system. Therefore, within the limitation of the present study, the types of implants and laser handpieces did not affect soft tissue healing and the osseointegration of implants.

## Figures and Tables

**Table 1:** Comparison of the bone volume/tissue volume (BV/TV) ratio (%) between the experimental and control groups.

Site	BV/TV Ratio (%)				*t*	*p* *
	Control Group		Experimental Group			
	Mean	SD	Mean	SD		
Proximal	2.85	0.34	3.14	0.16	−1.766	0.115
Distal	2.85	0.40	2.95	0.49	−0.351	0.734
Total	2.85	0.35	3.05	0.36	−1.243	0.230

* Significance determined using independent *t*-test, *p* < 0.05.

**Table 2:** Comparison of the bone-to-implant contact (BIC) ratio (%) between the experimental and control groups.

Site	BIC Ratio (%)				*t*	*p* *
	Control Group		Experimental Group			
	Mean	SD	Mean	SD		
Proximal	52.34	8.33	51.32	6.54	0.214	0.836
Distal	45.22	5.39	38.05	8.13	1.644	0.144
Total	48.78	7.61	44.69	9.87	1.039	0.312

* Significance determined using independent *t*-test, *p* < 0.05.

**Table 3:** Comparison of the bone-to-implant contact (BIC) distance (µm) between the experimental and control groups.

Site (Side of Rabbit Tibia)	BIC Distance (µm)				*t*	*p* *
	Control Group		Experimental Group			
	Mean	SD	Mean	SD		
Proximal	94.54	16.26	99.28	7.09	−0.598	0.574
Distal	110.28	10.03	117.68	11.59	−1.08	0.312
Total	102.41	15.20	108.48	13.27	−0.951	0.354

* Significance determined using independent *t*-test, *p* < 0.05.

**Table 4:** Comparison of the implant stability quotient (ISQ) values for different implant handpiece systems.

Group	ISQ Value (Mean ± SD)		*t*	*p* *
	Immediately after Surgery	1 Month after Surgery		
Experimental group	70.1 ± 10.4	79.9 ± 4.9	−9.527	<0.001 *
Control group	65.2 ± 11.6	79.0 ± 4.5	−7.018	<0.001 *
t	−2.458	−1.018		
*p* *	0.015 **	0.311 **		

* Significance determined by paired *t*-test, *p* < 0.05. ** Significance determined by independent *t*-test, *p* < 0.05.

**Table 5:** Comparison of the bone-to-implant contact (BIC) ratio (%) between the control and experimental groups.

Site (Side of Rabbit Tibia)	BIC Ratio (%)				*t*	*p* *
	Control Group		Experimental Group			
	Mean	SD	Mean	SD		
Proximal	53.90	0.00				
Distal	61.64	14.33	55.52	8.33	−0.55	0.656
Total	59.06	11.07	55.52	8.33	−0.44	0.682

* Significance determined using independent *t*-test, *p* < 0.05.

## Data Availability

The datasets used and/or analyzed during the current study are available from the corresponding author upon reasonable request.
